# Supplementary material for: DMRforPairs: identifying Differentially Methylated Regions between unique samples using array based methylation profiles
Source: BMC Bioinformatics. 2014 May 15;15:141. doi: 10.1186/1471-2105-15-141 (PMC4046028; doi:10.1186/1471-2105-15-141)
Supplement: Additional file 2 — DMRforPairs output for the comparison of A431-MCF7 and NA17018-NA17105. Please start from the HTML files in each folder. Available via the BMC Bioinformatics website. [file 1471-2105-15-141-S2.zip › 1394847754114233_MOESM2_ESM/A431_MCF7/figures/17508.pdf]

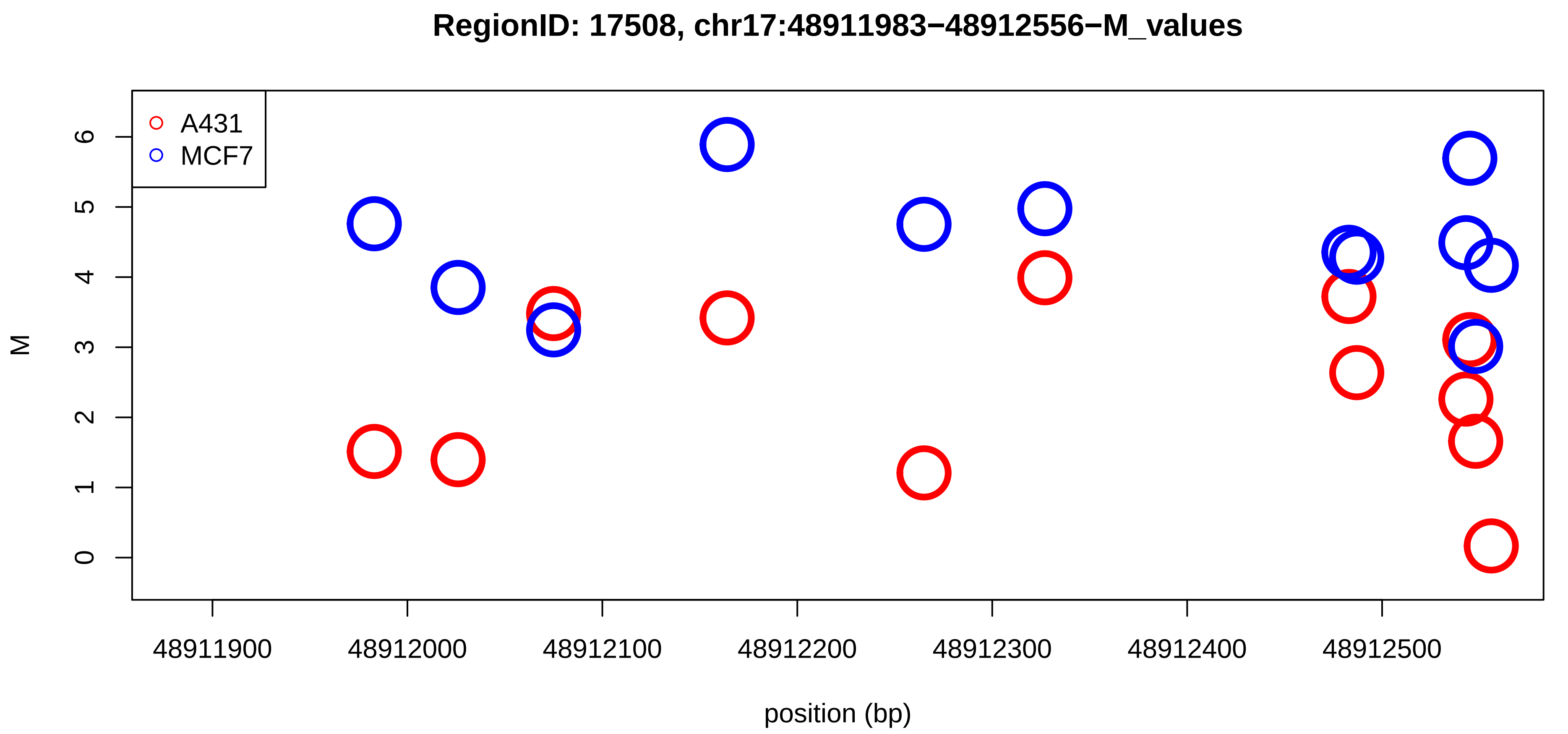
RegionID: 17508, chr17:48911983−48912556−M_values
A431
MCF7
M
0
1
2
3
4
5
6
48911900
48912000
48912100
48912200
48912300
48912400
48912500
position (bp)

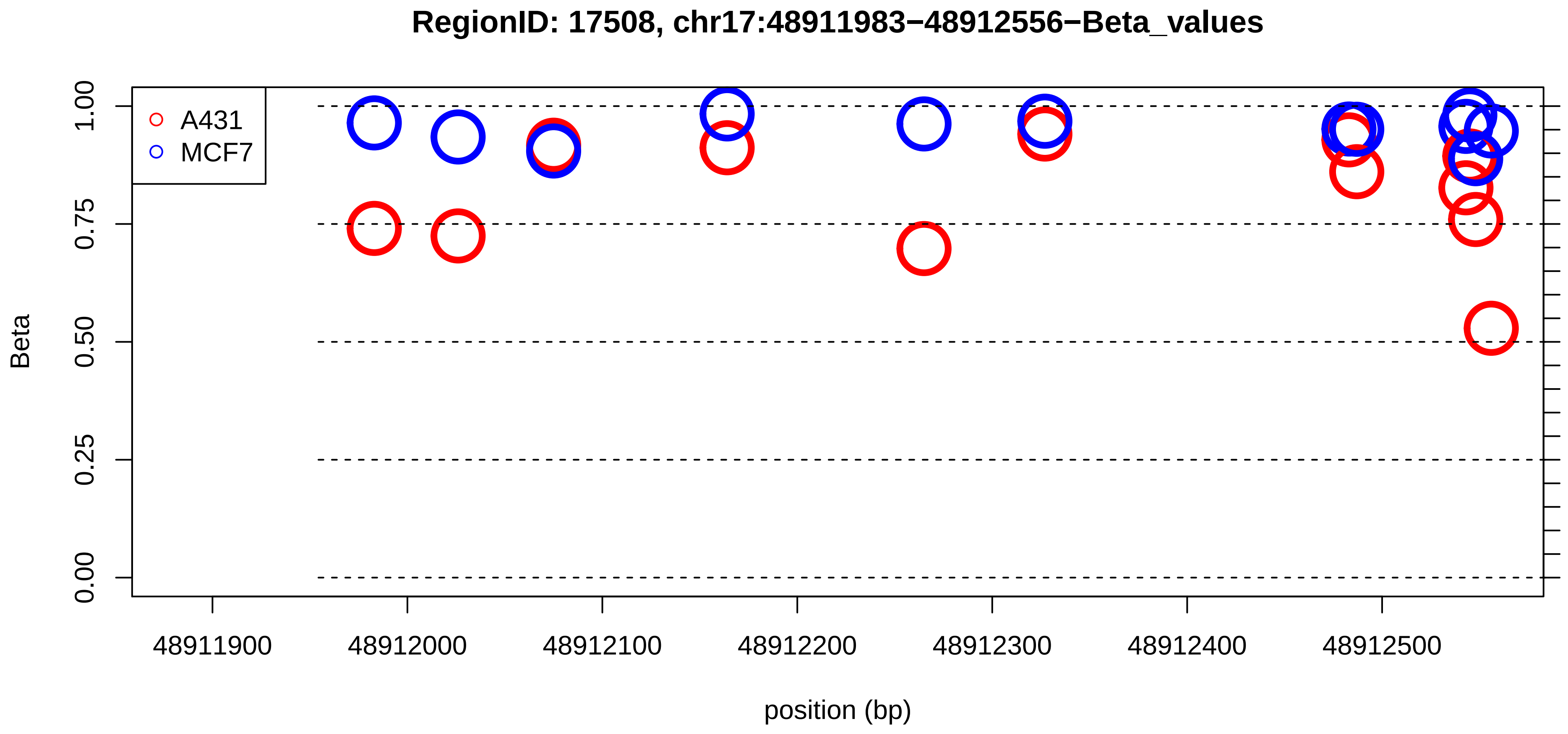
RegionID: 17508, chr17:48911983−48912556−Beta_values
A431
MCF7
Beta
0.00
0.25
0.50
0.75
1.00
48911900
48912000
48912100
48912200
48912300
48912400
48912500
position (bp)

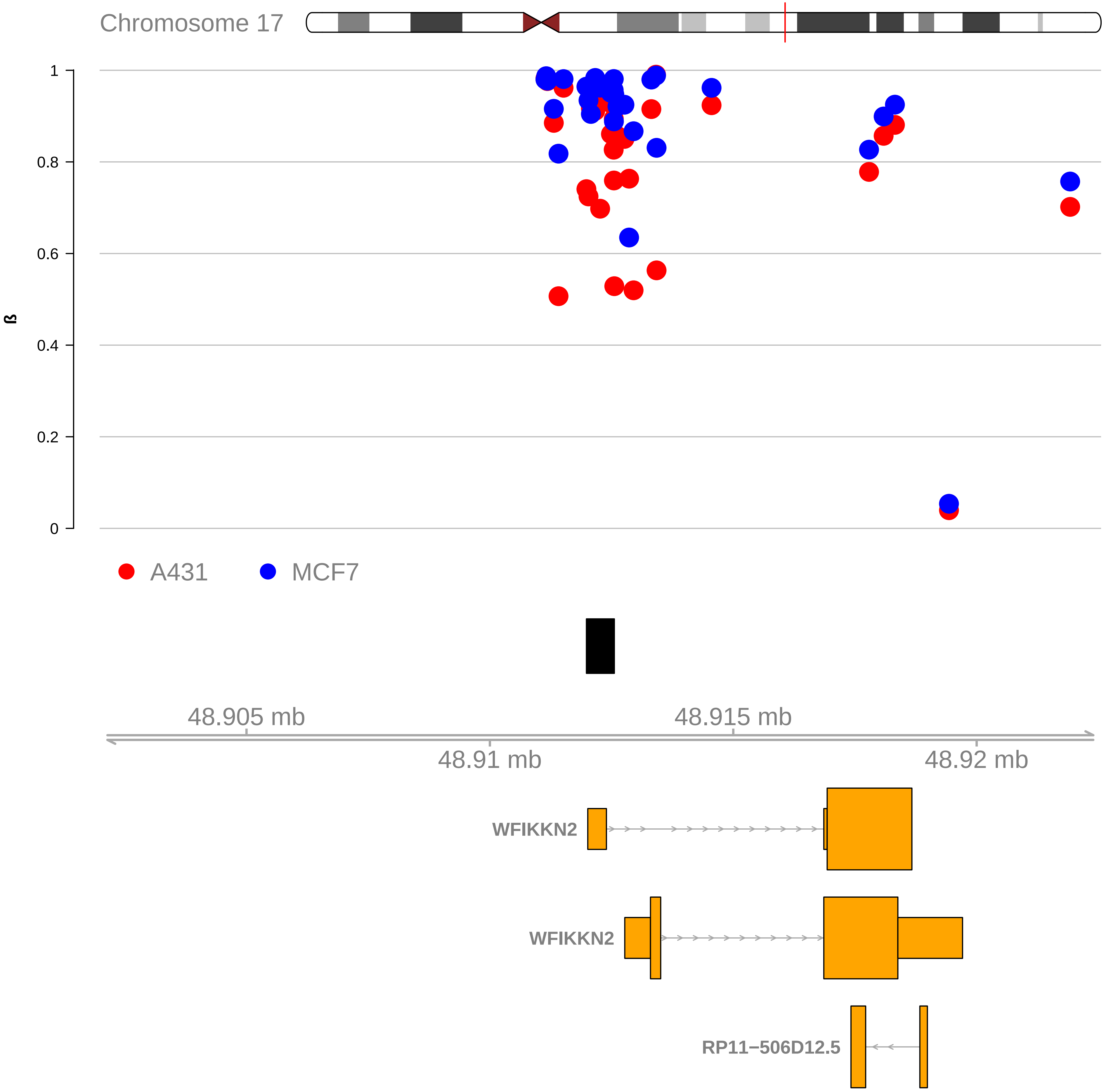

Chromosome 17
1
0.8
0.6
0.4
0.2
0
ß
A431
MCF7
48.905 mb
48.915 mb
48.91 mb
48.92 mb
WFIKKN2
WFIKKN2
RP11−506D12.5
